# A Validated RP-HPLC Method for Simultaneous Estimation of Nebivolol and Hydrochlorothiazide in Tablets

**DOI:** 10.4103/0250-474X.45420

**Published:** 2008

**Authors:** S. N. Meyyanathan, S. Rajan, S. Muralidharan, Arunadevi S. Birajdar, B. Suresh

**Affiliations:** Department of Pharmaceutical Analysis, J. S. S. College of Pharmacy, Ootacamund-643 001, India

**Keywords:** RP-HPLC method, simultaneous estimation, nebivolol, hydrochlorothiazide

## Abstract

A simple, selective, rapid, precise and economical reverse phase high pressure liquid chromatographic method has been developed for the simultaneous estimation of nebivolol and hydrochlorthiazide from pharmaceutical formulation. Phenomenex Gemini C_18_ (25 cm×4.6 mm i.d., 5 μ) column with a mobile phase consisting of acetonitrile: 50mM ammonium acetate (adjusted to pH 3.5 using orthophosphoric acid) (70:30 v/v) at a flow rate of 1.0 ml/min was used. Detection was carried out at 254 nm. Probenecid was used as an internal standard. The retention times of probenecid, nebivolol and hydrochlorthiazide were 13.05, 3.32 and 4.25 min, respectively. The developed method was validated in terms of accuracy, precision, linearity, limit of detection, limit of quantitation and solution stability. The proposed method can be used for the estimation of these drugs in combined dosage forms.

Nebivolol is chemically designated as 1-(6-fluorochroman-2-yl)-{[2-(6-fluorochroman-2-yl)-2-hydroxy-ethyl]amino}ethanol^1^. It is a selective β1 receptor blocker used in the treatment of hypertension. Hydrochlorothiazide belongs to the thiazide class of diuretics. Many methods have been described in the literature for the determination of nebivolol and hydrochlorothiazide individually and in combination with other drugs^2^–^15^. The present work describes the development of a validated RP-HPLC method, which can quantify these components simultaneously from a combined dosage form and the method, was validated following the ICH guidelines^16^.

Acetonitrile HPLC grade was procured from E. Merck (India) Ltd, Mumbai. Ammonium acetate AR grade was procured from Qualigens fine chemicals, Mumbai. Water HPLC grade was obtained from a Milli-QRO water purification system. Reference standards of nebivolol and hydrochlorothiazide were obtained from Unichem Pharmaceuticals, Mumbai and probenecid was procured from Cadila Pharmaceuticals Ltd, Ahmedabad.

Chromatographic separation was performed on a Shimadzu^^®^^ liquid chromatographic system equipped with a LC-10AT-vp solvent delivery system (pump), SPD M-10AVP photo diode array detector, Rheodyne 7725i injector with 50 μl loop volume. Class-VP 6.01 data station was applied for data collecting and processing (Shimadzu, Japan). A Phenomenex Gemini C_18_ column (25 cm×4.6 mm i.d., 5 μ) was used for the separation, mobile phase of a mixture of acetonitrile and 50 mM ammonium acetate (adjusted to pH 3.5 using orthophosphoric acid); (70:30 v/v) was delivered at a flow rate of 1.0 ml/min with detection at 254 nm. The injection volume was 50 μl and the analysis was performed at ambient temperature.

Standard stock solutions of 1.0 mg/ml nebivolol and hydrochlorothiazide were prepared separately using a mixture of water and acetonitrile (1:1 v/v) solvent. From the standard stock solution, mixed standard solutions were prepared with solvent of different concentrations ranging from 2 to 10 μg/ml of nebivolol and from 2.5 to 12.5 μg/ml of hydrochlorothiazide with 50.0 μg/ml of probenecid as internal standard.

Twenty tablets of Nebicard-H each containing 5.0 mg of nebivolol and 12.5 mg of hydrochlorothiazide were weighed and finely powdered; a quantity of powder equivalent to 5.0 mg of nebivolol and 12.5 mg of hydrochlorothiazide was weighed and transferred to a sintered glass crucible. To this 5.0 ml of 1.0 mg/ml solution of probenecid was added and the drugs were extracted with three quantities, each of 20 ml of mixture solvent. The combined extracts were made up to 100 ml with mobile phase and further dilutions were made to get a concentration of 5.0 μg/ml of nebivolol, 12.5 μg/ml of hydrochlorothiazide (theoretical value) and 50.0 μg/ml of probenecid as internal standard and this solution was used for the estimation.

With the optimized chromatographic conditions, a steady baseline was recorded, the mixed standard solution was injected and the chromatogram was recorded. The retention times of probenecid, nebivolol and hydrochlorothiazide were found to be 13.05, 3.32 and 4.25 min, respectively. This procedure was repeated for the sample solution obtained from the formulation. The response factor (peak area ratio of standard peak area to the internal standard peak area) of the standard solution and sample solution were used for calculation.

The HPLC procedure was optimized with a view to develop precise and stable assay method. The typical chromatogram of sample solution is shown in fig. 1. Detection was done at 254 nm. The peak area ratios of standard and sample solutions were calculated. The resolution factor at the above said condition was 1.7. The assay procedures were repeated for six times and mean peak area and mean weight of standard drugs was calculated and found to be 4.95±1.24 mg of nebivolol and 11.64±0.412 mg of hydrochlorothiazide. The results of analysis shows that the amounts of drugs were in good agreement with the label claim of the formulation.

**Fig 1:**
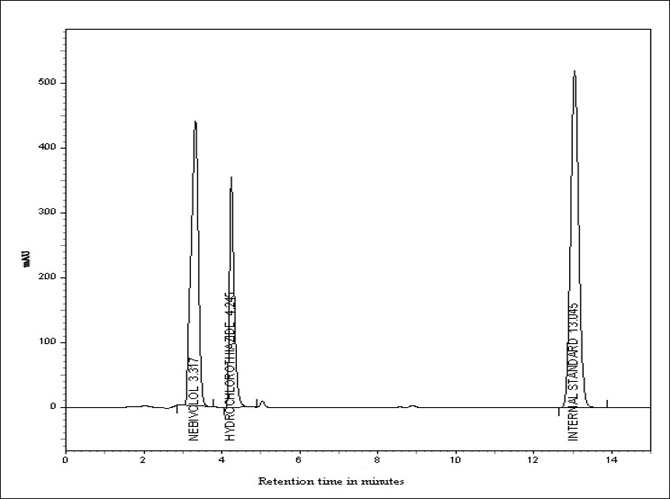
Typical chromatogram of sample solution Peak at 3.32 min is for nebivolol HCl, peak at 4.25 min is for hydrochlorothizide and peak at 13.05 min is for the internal standard, probenecid

The method was validated for accuracy, precision, linearity, LOD, LOQ, ruggedness and robustness as per ICH guidelines. The accuracy of the method was determined by recovery experiments. Recovery studies were carried out six times and the average recovery found to be 98.25% for nebivolol and 98.65% hydrochlorothiazide, respectively. The precision of the method was demonstrated by inter day and intra day variation studies and having RSD value less than 1%.

The linearity of the method was determined at five concentration levels ranging from 2 to 10μg/ml for Nebivolol and 2.5 to 12.5μg/ml for Hydrochlorothiazide. The slope and intercept value for calibration curve was y= 0.0026x−0.0025 (R^2^=0.998) for nebivolol and y= 0.0058x−0.0002 (R^2^=0.9995) for hydrochlorothiazide. The LOD for nebivolol and hydrochlorothiazide was found to be 10 ng/ml and 5.0 ng/ml, respectively. The LOQ was 30 ng/ml and 15 ng/ml for nebivolol and hydrochlorothiazide, respectively.

The ruggedness of the method was determined by carrying out the experiment on different instruments like Shimadzu HPLC (LC 2010 A HT), Agilent HPLC and Water's Breeze HPLC by different operators using different columns of similar type like Hypersil C_18_, Phenomenex Gemini C_18_ and Hichrom C_18_. Robustness of the method was determined by making slight changes in the chromatographic conditions. It was observed that there were no marked changes in the chromatograms, which demonstrated that the RP-HPLC method developed, are rugged and robust.

In order to demonstrate the stability of both standard and sample solutions during analysis, both solutions were analyzed over a period of 5 h at room temperature. The results show that for both solutions, the retention time and peak area of NEB and HCTZ remained almost unchanged (% RSD less than 2.0) and no significant degradation within the indicated period.

The column efficiency, resolution and peak asymmetry were calculated for the standard solutions (Table 1). The values obtained demonstrated the suitability of the system for the analysis of this drug combination and the system suitability parameters fall within ±3% standard deviation range during routine performance of the method.

**TABLE 1:** SYSTEM SUITABILITY STUDIES

Parameters	Nebivolol	Hydrochlorothiazide
Theoretical plate/meter	4525	5249
Resolution factor	1.75	1.75
Asymmetric factor	0.92	1.04
LOD (ng/ml)	10	5.0
LOQ (ng/ml)	30	15

The proposed RP-HPLC method for the simultaneous estimation of Nebivolol and Hydrochlorthiazide in combined dosage form is accurate, precise, linear, rugged, robust, simple and rapid. Hence the present RP-HPLC method is suitable for the quality control of the raw materials, formulations and dissolution studies.
